# Analysis of the frequency of single nucleotide polymorphisms in cytokine genes in patients with New Onset Diabetes After Transplant

**DOI:** 10.1038/s41598-021-84400-9

**Published:** 2021-03-16

**Authors:** Mohamed Jahromi, Torki Al-Otaibi, Osama Ashry Gheith, Nashwa Farouk Othman, Tarek Mahmoud, Parasad Nair, Medhat A-Halim, Parul Aggarwal, Grace Messenger, Philip Chu, Sacha A. De Serres, Jamil R. Azzi

**Affiliations:** 1 Clinical Research, Medical Division, Dasman Diabetes Institute, Kuwait City, Kuwait; 2 Nephrology Department, Hamad Al-Essa Organ Transplantation Center, Kuwait City, Kuwait; 3 Urology and Nephrology Center, Mansoura University, Mansoura, Egypt; 4 Community department, Faculty of Nursing, Manoura University, Mansoura, Egypt; 5 Education, Clinical Services Division, Dasman Diabetes Institute, Kuwait City, Kuwait; 6 Podiatry Department, Dasman Diabetes Institute, Kuwait City, Kuwait; 7 Vontz Center, University of Cincinnati, Cincinnati, USA; 8 CHU de Québec - Université Laval, Quebec, QC Canada; 9 Kidney Division, Transplantation Research Center, Harvard Medical School, Brigham and Women’s Hospital, Boston, USA; 10 Sehatek Awal, Manama, Bahrain

**Keywords:** Adaptive immunity, Diabetes complications

## Abstract

New Onset Diabetes After Transplantation (NODAT) is a serious metabolic complication. While β-cell dysfunction is considered the main contributing factor in the development of NODAT, the precise pathogenesis is not well understood. Cytokines are thought to be involved in the inflammation of islet β-cells in diabetes; however, few studies have investigated this hypothesis in NODAT. A total of 309 kidney transplant recipients (KTRs) were included in this study. An association between kidney transplants, and the development of diabetes after transplant (NODAT) was investigated. Comparison was made between KTRs who develop diabetes (NODAT cases) or did not develop diabetes (control), using key cytokines, IL-6 G (− 174)C, macrophage mediator; IL-4 C (− 490)T, T helper (Th)-2 cytokine profile initiator; Th-1 cytokine profile initiator interferon-γ T (+ 874) A gene and TGF β1 C (+ 869) T gene polymorphisms were investigated. The genes were amplified using well-established polymerase chain reaction (PCR) techniques in our laboratory. Compared to the AA and AT genotypes of interferon gamma (IFNG), there was a strong association between the TT genotype of IFNG and NODAT kidney transplant recipients (KTRs) versus non-NODAT KTRs (*p* = 0.005). The AA genotype of IFNG was found to be predominant in the control group (*p* = 0.004). Also, significant variations of IL6 G (− 174) C, IL-4 C (− 590) T, interferon-γ T (+ 874) A gene and transforming growth factor β1 C (+ 869) T may contribute to NODAT. Our data is consistent with theTh-1/T-reg pathway of immunity. Further larger pan Arab studies are required to confirm our findings.

## Introduction

New Onset Diabetes After Transplantation (NODAT) is a serious and common complication following organ transplantation. It is a frustratingly complex disease that is associated with increased mortality and morbidity possibly due to high rates of cardiovascular disease and infection. These factors are the leading causes of death in kidney transplant recipients (KTRs)^1,2^. The rate of NODAT varies depending on the age and ethnicity of patients at the time of transplant. Also, the usage of immunosuppressive agents plays a role in the development of NODAT^3^. NODAT is diagnosed typically between three months to one-year post-transplant, when transplant recipients are receiving a stable maintenance dose of immunosuppressive drugs, are free from infection, and have stable graft function^1–3^. Different studies have reported variable rates of NODAT ranging from 2 to 53%^4^. NODAT was reported to occur in 4–25% of KTRs^5–7^, and is more commonly manifested in African- Americans and Hispanics in comparison to Caucasians and Asians^8^. Information about NODAT in the Arab populations is scarce, with the exception of few epidemiologic studies^9–11^ suggesting that 25–30% of KTRs eventually develop NODAT^9–12^. Specifically, the incidence rate of NODAT, following kidney transplantation, has been found in to be as high as 27% in Saudi Arabia^9^, 30% in Bahrain^10^, 22.2% in Egypt^11^, and 25.6% in Kuwait^12,13^.

Genetic predisposition to NODAT likely involves an inherited defect in the peripheral tolerance to T-cell infiltration along with inadequate insulin secretion^14–16^. There is clear evidence showing that the different stages of NODAT are discrete entities, and that progression from one stage to the next is dependent on immune regulation^16^. T-cells are important for orchestrating the immune response and can be categorized into subsets according to their phenotypic characteristics resulting from polarization of naïve T-cells (i.e. Th-1, Th-2 and regulatory T-cell; T-reg)^17–19^. Each T-cell subset has a unique functional role highlighted by their capacity to produce pro-inflammatory and anti-inflammatory cytokines in response to various immune challenges. Cytokines are a group of pharmacologically active polypeptides that possess autocrine, paracrine, and juxtacrine effects with characteristic features^19^. Given the critical role of cytokines in regulating immune responses, subtle differences in cytokine expression may have a major effect on the outcomes of those responses^20^. Single nucleotide polymorphisms (SNP) in the regulatory region of the cytokine genes have been shown to correlate with individual variations in cytokine production^9–23^. Thus, variations in susceptibility to NODAT may be influenced by inter-individual genetic variations of cytokine genes^19–23^. Research on the association of SNPs have now become a potential means not only for better understanding the etiopathogenesis of the disease, but also as a probable marker for disease susceptibility and severity. It has been shown that interleukin-6(IL-6) derived from macrophages play a key role in inflammation^19,24^. This process is regulated via two major arms of the immune system: intra-cellular suppressor of cytokine signaling (SOCS) and T-reg cells^25^.

IL-6 gene (Online Mendelian Inheritance in Man #147620) maps to chromosome 7p21^26^. IL-6 a glycoprotein composed of 212 amino acids with a signal peptide of 27 amino acids and two potential NH2-linked glycosylation sites^26^. The molecular weight ranges from 21 to 28 kDa. IL-6 is a key cytokine with pleiotropic interactions in different human diseases pathogenesis^19^. IL-6 activates the immune system and enhances inflammatory response but also carries anti-inflammatory properties^27^. Understanding such pleiotropic effects of IL-6 in in the pathogenesis of the disease may help determine the progression, severity and duration of the disease. IL-6 is currently considered an important target for clinical interventions^28,29^. Hence, better understanding of IL-6 role in the pathogenesis of NODAT can provide rational and therapeutic intervention^29^. The guanine (G)/cytosine (C) polymorphism in the IL-6 proximal promoter region, at position IL-6 G (− 174) C, regulates transcription of the IL-6 gene and subsequently in IL-6 levels of plasma and serum^19,30^; the same polymorphism was found to be associated with fasting insulin levels,insulin sensitivity, and the integrated area under the curve of serum glucose concentrations^30,31^.

Interferon-gamma (IFN-γ), also known as type II interferon or macrophage-activating factor^32^, is a multipotent cytokine with an approximate molecular weight of 17 kDa. IFN-γ is secreted by activated T-cells and natural killer cells (NK), and it modulates many facets of the hosts immune response^33^. It enhances the hosts defense and innate immune response through promoting inflammation via the JAK-STAT1 signaling pathway and inhibiting both interleukin (IL)-4 (the initiator of the Th-2 cytokine cascade) and the promoter of the Th-1 cytokine profile^22^. TheDNA sequence of the human IFN‐γ gene (GenBank AF330164) shows the presence of a variable‐length CA repeat in the first intron of the gene where allele 2 (12 CA repeats) produces a high level of IFN‐γ protein^18^. This sequence is immediately adjacent and correlated with allele T for thiamine of the polymorphism at position + 874^34^. It is well established that the IFN-γ gene polymorphism T (+ 874) A (IFNG) of the first intron is correlated with serum level of IFN-γ production and mRNA expression in vitro and in vivo; the TT genotype correlates with high levels of IFN-γ production, and TA and AA are correlated with intermediate and low production level^18,22,34–38^. This polymorphism coincides with a putative NF-κB binding site that may mediate high production of IFN-γ^39,40^. Importantly, IFN-γ is believed to play an important role in the autoimmune pathogenesis of type 1 diabetes^22,41^. Inhibition of the IFN-γ function in non-obese diabetic (NOD) mice using either IFN-γ-specific antibodies^42^ or soluble IFN-γ receptors (IFN-γ-R)^43^ reduced the incidence of spontaneous diabetes and also prevented the transfer of diabetes via splenocytes from NOD donor mice^44^. Furthermore, transgenic expression of IFN-γ by β-cells induced autoimmunity, resulting in overt diabetes in otherwise diabetes-resistant mice^44^. The resistance of IFN-γ-R deficient animals to CD4+ T-cell mediated diabetes was shown to be primarily due to a lack of IFN-γ-R expression by β-cells. Kuriya et al. demonstrated that IFN-γ-R deficiency has distinct effects on CD4+ compared with CD8+ T-cell mediated diabetes, and that IFN-γ may play a critical role in CD4+ T-cell mediated destruction of β-cells^45^.

IL-4 is a 20-KDa glycoprotein encoded by the IL-4 gene on chromosome 5q23.31. It is secreted by helper T-cells (CD4) type 2 (Th-2), by NK cells, and by cells of the innate immune system: mast cells, basophils, and eosinophils^46^. IL-4 regulates proliferation, apoptosis, gene expression, and differentiation in many hematopoietic cells; it directs the immunoglobulin (Ig0) class switch to IgG1 and IgE, downregulates the production of Th-1 cells and is a critical mediator of the Th 1/ Th-2 cytokine milieu counterbalance^22,46^. IL-4 appears to protect human islets from cytotoxic damage induced by proinflammatory and Th-1 cytokines. Another study showed that long-term exposure of rat pancreatic islets to IL-4 resulted in an inhibitory action to certain islet functions^47^. These phenomena occur by the modulation of the homing of autoreactive cells to inflammatory sites and the stabilization of a protective Th-2-mediated environment in the thymus, spleen, and pancreatic islets. Thus, IL-4 treatment favors the expansion of regulatory CD4+ Th-2 cells in vivo and prevents the onset of insulitis and type 1 diabetes mediated by autoreactive Th-1 cells^22,48^. It has been suggested that IL-4 protects human islets from cytotoxic damage induced by proinflammatory and Th-1 cytokines. The local expression of IL-4 in the pancreatic islets of NOD mice (ins-IL-4 mice) restricted the activation of autoreactive T-cells and promoted complete protection against spontaneous diabetes^49^. IL-4 T (− 590) C, rs2243250, is a functional promoter of gene polymorphism, where C is substituted by T^22^. It is well established that TT genotype of IL-4 T (− 590) C corresponds to high level of IL-4 serum and mRNA production in vivo as well as in vitro. The same is true for IL-4 C (− 590) C for low levels, and TC for putative intermediate protein levels^22,50^.

Transforming growth factor-β1 (TGF-β1) belongs to a family of multifunctional growth factors which have profound regulatory effects on many developmental and physiological processes^51^.The human TGF-β1 gene is located on chromosome 19q13.1–13.3^52^, and more than ten polymorphic loci are presently known that are distributed across exons, introns, and the 5′flanking region^53^. A single nucleotide polymorphism (SNP) of codon 10 in the TGF-β1 T (+ 869) C gene(TFGB), T (proline) to C (leucine) is associated with different diseases including type 1 and type 2 diabetes^54^. There is well-established evidence regarding the association of codon 10 with varying levels of TGF-β1 synthesis in vitro and in vivo^23^. Increases or decreases in the production of TGF-β1 have been linked to numerous diseases including atherosclerosis, and fibrotic diseases of the kidney, liver, and lung^23^.

There is increasing evidence to show that SOCS proteins may be involved in the development of diabetes and its associated complications^55^. In addition, SOCS-modulating properties have been attributed to pharmacological agents that are currently used for the treatment of diabetes^56^.

Our results indicate that susceptibility to NODAT might be monitored by genotyping KTRs who developed diabetes compared with KTRs who did not develop diabetes for selected major Th 1(IFNG)/ Th-2 (IL-4)/TGFB, T-reg in addition to IL-6, macrophage derived cytokines. We have investigated well-established SNPs in major cytokines, according to their site of origin, in order to identify our future line of research.

## Materials and methods

This piece of research was conducted following written approval from Dasman Diabetes Institute Research and Ethical Committee reference: RA 2015-013. Oral and written informed consent was obtained from all participants prior to data collection. The methods were performed in accordance with the relevant rules and regulations. This work was conducted in collaboration between Dasman Diabetes Institute and Hamed Al-Essa Organ Transplant Center of Kuwait. KTRs with NODAT and non-NODAT were recruited from May 2015 until March 2017. A total of 309 KTRs were recruited: 155 patients in the non-diabetic, non-NODAT cohort and 154 patients who developed NODAT following kidney transplantation (Table 1).

**Table 1:** Characteristics of studied subjects.

Age group/years	NODAT		CNTRL	
	N	%	N	%
< 40	28	18.1	98	63.2
40–60	80	52.0	45	29.1
> 60	46	29.9	12	7.7
Donor mean age/years	34.4 ± 9.1		34.7 ± 8.8	
**Nationality**				
Kuwaiti	76	49.4	93	60.0
Non-Kuwaiti	78	50.6	62	40.0
Patient sex(male/female)	107/47		112/43	
**Original kidney disease**				
Idiopathic	42	27.3	33	21.3
Glomerulonephritis	50	32.5	69	44.5
Hypertension	10	6.5	9	5.8
Urological	10	6.5	14	9.0
Others	42	27.2	30	19.3
Virology	+ve/−ve		+ve/−ve	
HCV	12/142		2/153	
CMV IgM	1/153		5/150	
IgG	153/0		148/5	
**Dialysis type**				
Hemodialysis	118	76.6	121	78.0
Peritoneal dialysis	16	10.4	14	9.1
Preemptive	20	13.0	20	12.9
Pre-transplant co-morbidities Hypertension	123	87.9	117	81.2
Pre-transplant TB exposure	41	26.6	41	26.5
Ischemic heart disease	19	14.0	13	9.0
Bone disease	28	20.4	27	19.3
Anemia	41	30.8	39	28.5
Hyperlipidemia	7	5.3	6	4.3
**Donor type**				
Live related	132	85.7	125	80.7
Cadaveric	22	14.3	30	19.3
**Mean HLA mismatch**				
A locus	1.10 ± 0.62		1.04 ± 0.63	
B locus	1.53 ± 0.98		1.22 ± 0.68	
DR locus	1.02 ± 0.71		0.97 ± 0.68	
Total	3.44 ± 1.46		3.26 ± 1.56	
**Induction immunosuppression**				
None	20	13.0	11	7.1
IL2 receptor blocker (simulect)	34	22.1	30	19.4
Anti-thymocyte globulin	47	30.5	69	44.5
Others	53	34.4	45	29.0
**Type of immunosuppression:**				
Cyclosporine based	82	53.9	63	42.0
Tacorlimus based	64	42.1	82	54.7
Steroid free	2	1.3	4	2.7
CNI free	4	2.6	1	0.7
**Graft function**				
Immediate	90	58.4	94	60.6
Slow graft function	21	13.6	36	23.2
Delayed graft function	9	5.8	6	3.9
Unknown	34	22.1	19	12.3
Basal BMI (mean ± SD)	28.07 ± 5.58		26.11 ± 7	
Last BMI(mean ± SD)	29.92 ± 5.37		29.04 ± 6.2	
Mean rejection episodes	1.32 ± 0.47		1.47 ± 0.66	
**Graft outcome**				
Functioning	140	91	145	94
Failed	12	8	6	4
Lost follow up	3	1	4	3
**Patient outcome**				
Living	150	97	151	97
Dead	2	1	0	
Lost follow up	2	1	4	3
Basal fasting blood sugar	9.5 ± 1.4		4.5 ± 0.65	
Basal HbA1C	6.96 ± 1.5		5.2 ± 0.46	

General characteristics of study groups and their distribution among NODAT and control.

Genomic DNA was extracted from a 5 ml sample of fresh peripheral blood using the QIAamp DNA Mini Kit (QIAGEN) according to the manufacturer’s instructions. Extracted DNA sample had a final concentration of 55–365 ng/ml, and samples were stored at −20°C prior to use. Allele-specific sequence primers were used to amplify IL-6G, IFNG, IL4G and TGFG using well-established techniques available in our laboratories (Table 2)^22^, and PCR products were separated on a 2% agarose gel. To ensure the quality of the experiments, 10% of all samples were genotyped twice, producing 100% reproducibility.

**Table 2:** Amplimers and PCR conditions.

(A) IFNG and TGFB	Amplimers		
IFNG-FP generic:5′-tcaacaaagctgatactcca-3’			
IFNG-RP A-allele:5′-ttcttacacaaaatcaaatca-3′;	IFNG-RP T-allele: 5′-ttcttacaacacaaaatcaaatct-3’		
TGFB-FP generic: 5′-tccgtgggatactgagacac-3'			
TGFB-RP C-allele: 5′-gcagcggtagcagcagcg-3′;	TGFB-RP T-allele: 5′-agcagcggtagcagcagca-3'		

(B) IFNG and TGFB PCR conditions		PCR mix − total volume20 µl	
PCR steps	Temp (°C)	Time	Cycles
Denaturation	95	1 min	–
Denaturation	95	15 s	10 cycles
Annealing	62	40 s	
Extension	72	40 s	
Denaturation	95	1 min	20 cycles
Annealing	54	1 min	
Extension	72	1 min	
Extension	72	5 min	

(C) IL-4 and IL-6 amplimers			
IL-4 FP:5′-gttgtaatgcagtcctcc-3′	IL-4RP:5′-actaggcctgatacg-3′		
IL-6 FP: 5′-ttgtcaagacatgccaaagtgc-3′	IL-6 RP:5′gggaaaatcccacatttgataa-3′		

(D) IL-4 and IL-6 PCR conditions		PCR mix − total volume25 µl	
PCR steps	Temp (°C)	Time	Cycles
Denaturation	95	1 min	–
Denaturation	95	1 min	30 cycles
Annealing	55	1 min	
Extension	72	1 min	
Extension	72	5 min	

Digestion of IL4 done by BSmFI 65 °C 15 min 0.1 µl of RE and 1 µl cutting buffer for 10 µl PCR sample.

Digestion of IL6 done by NIaIII 37 °C 15 min 0.02 µl of RE and 1 µl cutting buffer for 10 µl PCR sample.

List of amplimers and PCR conditions used to amplify the promoter cytokines of their categories.

### Diagnosis of NODAT

All kidney transplant recipients were screened for fasting plasma glucose and glycated hemoglobin, according to Kidney Disease: Improving Global Outcomes (KDIGO) guidelines^57^. Abnormal results were confirmed using an oral glucose tolerance test. In these cases, diabetes management was introduced immediately that included diet, exercise, oral agents and/or insulin, in addition to regular blood glucose monitoring at home.

### Immunosuppression protocol

The immunosuppression protocol consisted of five doses of anti-thymocyte globulin (Sanofi US, Bridgewater, NJ, USA) for high-risk patients (re-transplants, prior pregnancy, blood transfusion, HLA-antibody positive, and/or more than four HLA mismatches), or two doses of IL-2 receptor blocker (Basiliximab, Novartis, Inc., Switzerland) for low-risk patients. Maintenance therapy consisted of prednisolone, mycophenolic acid, and a calcineurin inhibitor (CNI). The dose of CNI was gradually decreased over 12 months guided by a 12-h trough level.

Acute cellular rejection was treated using intravenous methylprednisolone sodium succinate (solumedrol, 1 g daily for 3 days) or thymoglobulin (1 mg/kg for 7–10 days) for steroid-resistant rejection. Antibody-mediated rejection was treated using plasma exchange, intravenous immunoglobulin (2 g/kg), and rituximab. All rejection episodes were biopsy-proven according to the Banff criteria (2015)^58^. Patients who received thymoglobulin as anti-rejection therapy were managed by universal chemoprophylaxis for both cytomegalovirus (CMV) and Pneumocystis Jirovecii Pneumonia (PJP). Valganciclovir was used as CMV secondary prophylaxis for one month, while those who developed CMV viremia during this period were managed with a therapeutic dose for three weeks, followed by three months prophylaxis. Trimethoprim was used for one month as a prophylaxis for PJP. Associated infections were recorded if patients required hospital admission. Details of the patients who developed CMV infection or rejection episodes during the study period were recorded. Clinical data were collected with special emphasis on patient age, sex, donor type, immunosuppressive therapy, dialysis type and duration, primary kidney disease, pretransplant comorbidities, details of rejection episodes, post-transplant infections and graft and patient outcome.

### Statistical analysis

The Hardy–Weinberg equilibrium was assessed using a chi-square test based on a comparison of the observed and expected genotypes. Statistical analyses were performed using SPSS software (SPSS, version 20.0, IBM Corporation, Armonk, NY, USA). The sample size was calculated to accept a marginal error of 6.6% (95% confidence interval) in a normally-distributed population. Allelic and genotypic distribution of IL6G, IFNG, IL-4G and TGFBG between both cohorts were compared using a paired-sample t-test, independent sample t-test, chi-square test, Fisher’s exact test, and ANOVA, as appropriate. Results are expressed as mean ± standard deviation, and differences were considered significant at *p* < 0.05.

## Results

### Phenotypic features

The participants age ranged between 20 and 80 years, the mean age for recipients who developed NODAT was 52.85 ± 11.4 years compared to 38.97 ± 13.1years in the control group. Younger patients(<40years) were more prevalent in the control group, while patients older than 40 years were predominant in NODAT, *p* < 0.0001. All patients received grafts from donors aged 30–40 years. The mean donor age was 34.4 ± 9.1 in cases versus 34.7 ± 8.8 for controls (Table 1). We noticed that the two groups were homogenous in variability of original kidney disease, dialysis type, donor type, and type of immunosuppression (both induction and maintenance). As well as that, all subjects had no history of diabetes as demonstrated by normal fasting and postprandial blood glucose levels prior to transplantation.

There were no differences in ethnicity of our cohorts, 55% Kuwaiti, 45% non-Kuwaiti (Table 3). Moreover, no statistically significant differences were noticed in pre-transplant comorbidities in both cohorts, especially hypertension, history tuberculosis treatment, ischemic heart disease, bone disease, anemia, and hyperlipidemia, HLA class 1 -A and -B and class II DR, *p* > 0.05, Table 3. There was a significantly higher prevalence of patients with chronic hepatitis C virus (HCV) infection in NODAT (12 cases in NODAT compared to two cases in control, *p* = 0.006, Table 3). In addition, there were significantly more CMV IgG positive patients for in NODAT than control (153 vs. 148, respectively; *p* = 0.02). However, the two groups were comparable regarding pre transplant CMV IgM (*p* > 0.05). Evaluation of immediate post-transplant graft function showed that the number of patients with slow graft function was significantly higher in control (*p* = 0.03). Overweight, as measured by mean basal body mass index (BMI), was significantly higher in the NODAT group compared to the control group (28.07 ± 5.5 vs. 26.11 ± 7, respectively; *p* = 0.01), while follow-up mean BMI was comparable in both cohorts irrespective of age, *p* = 0.21, see (Table 3).

**Table 3:** Demographic data about NODAT and control.

Age group/years	NODAT		CNTRL		*p* value	Chi square	Odd ratio
	n = 154	%	n = 155	%			
21–< 40	28	18.1	98	63.2	< 0.0001	68.61	
40–60	80	52.0	45	29.1		58.10	
> 60	46	29.9	12	7.7	< 0.0001	68.62	
					< 0.0001		
Donor mean age/years	34.4 ± 9.1		34.7 ± 8.8		0.77	2.31	
**Nationality**							
Kuwaiti	76	49.4	93	60.0			
Non-Kuwaiti	78	50.6	62	40.0	0.060	3.5	0.65
Patient sex(male/female)	107/47		112/43		0.591	0.29	0.87
**Original kidney disease**							
Idiopathic	42	27.3	33	21.3	0.14	6.83	
Glomerulonephritis	50	32.5	69	44.5	0.10	6.10	
Hypertension	10	6.5	9	5.8	0.15	6.82	
Urological	10	6.5	14	9.0	0.146	4.60	
Others	42	27.2	30	19.3	0.132	6.83	
Virology	+ ve/-ve		+ ve/-ve				
HCV	12/142		2/153		0.006	7.55	0.155
CMV IgM	1/153		5/150		0.10	2.39	
IgG	153/0		148/5		0.024	7.08	2.03
**Dialysis type**							
Hemodialysis	118	76.6	121	78.0	0.157	5.20	
Peritoneal dialysis	16	10.4	14	9.1	0.154	5.26	
Preemptive	20	13.0	20	12.9	0.251	1.20	
**Pre-transplant co- morbidities**							
Hypertension	123	87.9	117	81.2	0.124	2.36	0.59
Pre-transplant TB exposure	41	26.6	41	26.5	0.97	0.001	0.99
Ischemic heart disease	19	14.0	13	9.0	0.194	1.69	
Bone disease	28	20.4	27	19.3	0.810	0.06	
Anemia	41	30.8	39	28.5	0.671	0.18	0.89
Hyperlipide mia	7	5.3	6	4.3	0.714	1.34	
**Donor type**							
Live related	132	85.7	125	80.7	0.28	1.42	1.44
Cadaveri c	22	14.3	30	19.3			
**Mean HLA mismatch**							
A locus	1.10 ± 0.62		1.04 ± 0.63		0.391		
B locus	1.53 ± 0.98		1.22 ± 0.68		0.172		
DR locus	1.02 ± 0.71		0.97 ± 0.68		0.630		
Total	3.44 ± 1.46		3.26 ± 1.56		0.350		
**Induction immunosuppression**							
None	20	13.0	11	7.1	0.05	6.44	
IL2 receptor blocker (simulect)	34	22.1	30	19.4	0.05	6.77	
Anti-thymocyte globulin	47	30.5	69	44.5	0.05	7.68	
Others	53	34.4	45	29.0	0.05	6.65	
**Type of immunosuppression:**							
Cyclosporine based	82	53.9	63	42.0	0.067	7.16	
Tacorlimus based	64	42.1	82	54.7	0.062	7.31	
Steroid free	2	1.3	4	2.7	0.065	6.99	
CNI free	4	2.6	1	0.7	0.067	6.2	
**Graft function**							
Immediate	90	58.4	94	60.6	0.9	1.22	
Slow graft function	21	13.6	36	23.2	0.03	8.87	
Delayed graft function	9	5.8	6	3.9	1.0	2.33	
Unknown	34	22.1	19	12.3	0.02	8.98	
Basal BMI (mean ± SD)	28.07 ± 5.58		26.11 ± 7		0.014		
Last BMI(mean ± SD)	29.92 ± 5.37		29.04 ± 6.2		0.215		
**BK virus infection**							
BK viremia	12 (9)		21 (14)		0.12	2.3	1.7
BK Nephropathy	3 (2)		2 (1)		0.63	0.23	0.64
CMV viremia	21 (19)		40 (34)		0.01	6.3	2.1
Mean rejection episodes	1.32 ± 0.47		1.47 ± 0.66				
**Graft outcome**							
Functioning	140 (91)		145 (94)		0.4		
Failed	12 (8)		6 (4)		0.14		
Lost follow up	3 (1)		4 (3)		0.71	2.7	
**Patient outcome**							
Living	150 (97)		151 (97)		1		
Dead	2 (1)		0		0.24		
Lost follow up	2 (1)		4 (3)		0.68	2.66	
Basal fasting blood sugar	9.5 ± 1.4		4.5 ± 0.65		0.001		
Basal HbA1C	6.96 ± 1.5		5.2 ± 0.46		0.001		

Demographic features of NODAT and control. Young participants, > 40 years old, individuals were significantly more frequent among controls, *p* ≤ 0.0001. The frequencies of NODAT in 40–60 and > 60 years old groups were higher than control, *p* < 0.0001 for both (pathophysiological feature of our NODAT).

Induction of immunosuppressive drugs were significantly associated with NODAT than controls, *p* = 0.05 for all.

Slow graft function showed to be more frequent in controls than NODAT, *p* = 0.03.

Both basal fasting glucose and HbA1C were significantly higher in NODAT than control, *p* = 0.001.

The number of cases with post-transplant CMV viremia was significantly higher in control compared to NODAT (*p* = 0.01, Table 4). Moreover, there was no significant difference between NODAT and control groups regarding both graft and patient outcome (*p* > 0.05).

**Table 4:** Virology compared with frequencies of IL-6, IL-4, IFNG and TGFB in NODAT versus control.

Virology	NODAT		Control		P value	Chi Square	OR
	n	%	n	%			
**HCV positivity**							
GG	3	4.4	0		0.13	2.26	
IL-6 CG	8	15.5	1	1.8	0.013	6.19	
CC	1	3.2	1	2.1	0.752	0.10	
**Post-Tx CMV viremia**							
GG	14	25.5	14	46.7	0.02	5.6	3.7
IL-6 CG	5	13.5	13	27.7	0.117	2.5	
CC	2	11.8	13	32.5	0.10	2.6	
**HCV positivity**							
TT	3	12.5	2	4.1	0.181	1.8	
IL-4 TC	7	9.3	0		0.006	7.4	
CC	2	3.6	0		0.291	1.1	
**Post-Tx CMV viremia**							
TT	4	25.0	11	28.9	0.76	0.1	
IL-4 TC	11	20.4	23	44.2	0.009	6.9	
CC	6	15.4	6	22.2	0.47	0.5	
**HCV positivity**							
AA	2	9.1	0		0.05	3.8	
IFNG AT	6	12.2	0		0.007	7.4	
TT	4	4.8	2	3.5	0.70	0.1	
**Post-Tx CMV viremia**							
AA	4	30.8	10	38.5	0.63	0.2	
IFNG AT	7	18.0	12	25.0	0.50	0.4	
TT	10	16.9	18	41.9	1.0		
**HCV positivity**							
CC	3	15.0	1	2.3	0.09		
TGFB TC	3	7.0	0		0.11		
TT	6	6.7	0		0.08		
**Post-Tx CMV viremia**							
CC	2	14.3	8	26.7	0.46		
TGFB TC	3	11.1	15	37.5	0.02		
TT	16	23.9	15	36.6	0.19		

IL-6 CG was more frequent in NODAT than control, *p* = 0.03; while IL-6 GG showed to be more frequent among control than NODAT, *p* = 0.003 in post-transplant CMV.

IL-4 TC also was more frequent in NODAT than control, *p* = 0.006.

On the other hand, IL-4 TC was more frequent on control than NODAT, *p* = 0.009.

IFNG AA and AT were more frequent among NODAT compared to control in HCV positive individuals, *p* = 0.05, 0.007 respectively.

While post-transplant CMV was not associated to the disease, TGFB did not show any statistically significant association with NODAT nor control in HCV positive individuals.

While TGFB TC was significantly more in control subjects, *p* = 0.02.

### Frequencies of cytokine genotypes according to protein production

**Figure 1:**
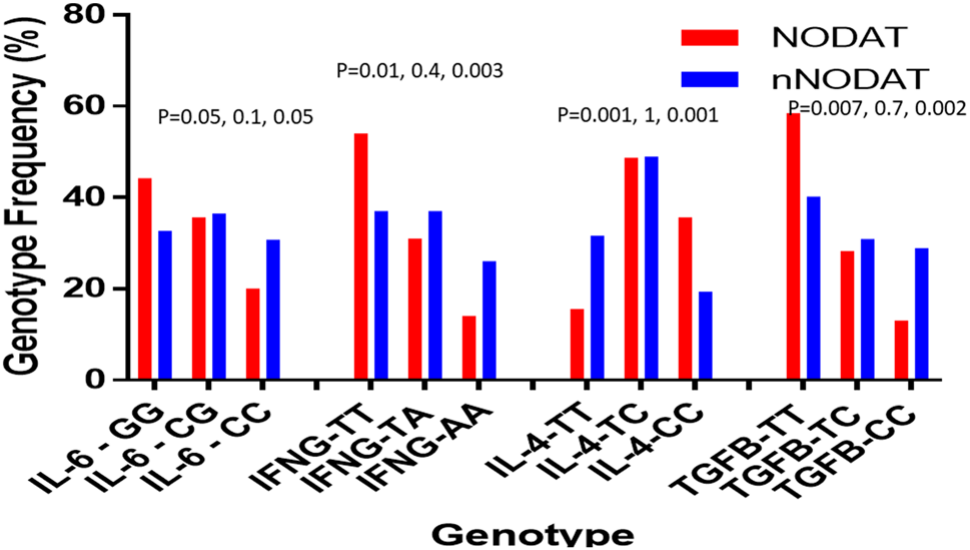
Genotypic frequencies of cytokines in NODAT and controls. Genotypes of cytokines are illustrated as homozygote (corresponded to high protein producer), heterozygote (corresponded to intermediate protein producer) and homozygote (corresponded to low protein producer). IL-6, IFNG and TGFB high producer genotypes were strongly associated with the disease, *p* = 0.05, 0.01 and 0.007; while, IL-4 high producer corresponded genotype was strongly associated with control cases, *p* = 0.001. In all cytokines intermediate protein level genotype did not show any significant association. Low protein producer corresponded genotypes were strongly associated with control group in IL-6, IFNG and TGFB, *p* = 0.05, 0.003 and 0.002; while, low protein level corresponded genotype in IL-4 was significantly associated with the disease, *p* = 0.001.

Patients with of IL-6(CC), IFN-G (AA), and TGF-B (CC), corresponding to low protein production, were significantly higher in control (*p* = 0.05, *p* = 0.01, and *p* = 0.001, respectively, Fig. 1). IL-6(GG), IFN-G (TT), and TGF-B (TT) genotypes, correlating to high protein production, were significantly higher in NODAT (*p* = 0.05, *p* = 0.003 and *p* = 0.002, respectively). On the other hand, patients with IL-4 genotype (TT) that correspond to high IL-4 production were significantly higher in the control group while the low producers were significantly higher in NODAT, *p* = 0.001.

### Frequencies of cytokine genotypes and virology

Genotype frequencies of IL-6(CG), IL-4(TC) and IFNG (AA, AT) were found to be significantly more prevalent among HCV positive patients in NODAT group (*p* = 0.013, *p* = 0.006, *p* = 0.05 and *p* = 0.007 respectively, Table 4). Genotype frequencies of IL-6(GG), IL-4(TC), TGF-B(TC) were found to be significantly more prevalent amongst patients with post-transplant CMV viremia in the control group (*p* = 0.02, *p* = 0.009 and *p* = 0.02 respectively, Table 4). Importantly, no significant association was noticed with IFNG genotypes, Table 4.

### Age specificity and genotypic frequency

**Figure 2:**
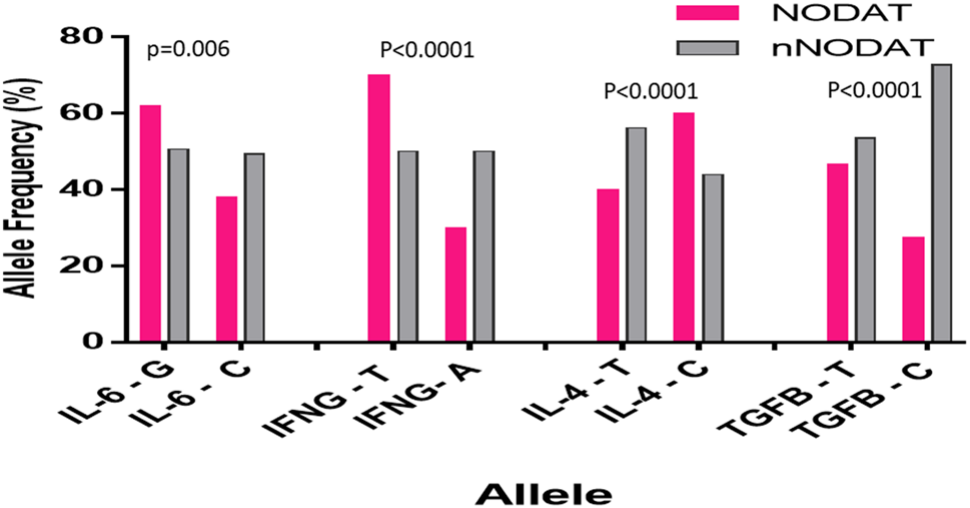
Allelic frequencies of cytokines in NODAT and controls. Allelic distribution showed to be significant in NODAT compared to control, IL-6 (*p* = 0.006), IL-4 (*p* ≤ 0.0001), IFNG (*p* ≤ 0.0001) and TGFB (*p* ≤ 0.0001).

The allelic distribution of the IL-6, IL-4, IFNG and TGFB among both studied cohorts were significant, *p* = 0.006, *p* < 0.0001, *p* = 0.0002 and *p* < 0.0001 (Fig. 2) however, these effects were only observed when the alleles were paired in genotypes.

Genotype frequencies of IL-6, IL-4, IFNG and TGFB did not show any statistical gender specificity among NODAT versus control. IL-6 GG gene frequency was predominant in control participants < 40 years of age, *p* < 0.0001, while it was most frequent in patients 40-60 years of age in the NODAT group, *p* = 0.02. Although its distribution was greater in patients > 60 years of age in the NODAT group, this data did not rank to significance, *p* = 0.06 (Table 5). IL-6 GC was predominantly more frequent in control patients > 40 years of age versus NODAT, p=0.003 (Table 5), while its frequency was shifted towards NODAT in patients > 60 years of age, *p* = 0.002. The same was true for IL-6 CC genotype distribution. Its frequency was more in the controls among > 40 years of age patients, *p* = 0.0001, and significantly increased in > 60 years of age NODATs, *p* = 0.005, Table 5. IL-4 TT was more abundant in the controls among patients < 40 years of age when compared to NODAT, *p* = 0.01. However, TT distribution was comparable in 40-60 years of age groups. No statistically significant results were found between control and NODAT in the > 60 years of age group and this is likely due to the small number of cases, Table 4.

**Table 5:** Age at onset compared with frequencies of IL-6, IL-4, IFNG and TGFB in NODAT versus control.

Age group & genotypic frequencies/years		NODAT	%	Control	%	P value	Chi Square	Odd Ratio
**IL-6**								
GG	< 40	9	13.2	26	52.0	< 0.0001	20.8	0.1
	40–60	35	51.5	15	30.0	0.03	5.4	2.4
	> 60	24	35.3	9	18.0	0.06	4.3	2.5
	< 40	13	23.6	34	59.6	0.0001	14.2	4.8
CG	40–60	29	52.7	21	36.8	0.13	1.1	1.9
	> 60	13	23.6	2	3.5	0.002	9.8	8.5
	< 40	16	19.4	38	79.2	0.0001	14.9	0.2
CC	40–60	16	51.6	9	18.8	0.06	4.5	8.5
	> 60	9	29.0	1	2.1	0.005	–	–
**IL-4**								
TT	< 40	8	33.3	32	65.3	0.01	6.7	0.3
	40–60	13	54.2	15	30.6	0.07	3.8	2.5
	> 60	3	12.5	2	4.1	0.03	0	
	< 40	11	14.7	46	60.5	< 0.0001	33.8	0.1
TC	40–60	34	45.3	22	28.9	0.04	4.3	2.9
	> 60	30	40.0	8	10.5	< 0.0001	17.4	8.9
	< 40	9	16.4	20	66.7	< 0.0001	21.9	0.2
CC	40–60	33	60.0	8	26.7	0.006	8.6	2.0
	> 60	13	23.6	2	6.7	0.07	3.9	0.1
**IFNG**								
AA	< 40	5	23.0	30	73.0	0.0002	14.8	0.1
	40–60	10	46.0	8	20.0	0.04	4.7	3.4
	> 60	7	32.0	3	7.0	0.02	–	
	< 40	8	16.0	36	63.0	< 0.0001	23.8	0.1
AT	40–60	2	9.6	19	33.0	0.01	7.1	2.9
	> 60	12	25.0	2	4.0	0.002	10.1	8.9
	< 40	15	18.0	32	56.0	< 0.0001	22.0	0.2
TT	40–60	41	49.0	18	32.0	0.06	4.2	2.0
	> 60	27	33.0	7	12.0	0.03	5.1	0.1
**TGFB**								
CC	< 40	2	10.0	30	69.8	< 0.0001	19.5	0.1
	40–60	11	55.0	11	25.6	0.03	5.2	3.6
	> 60	7	35.0	2	4.7	0.002	3.6	11.0
	< 40	9	20.9	26	56.5	0.001	11.8	0.2
TC	40–60	21	48.8	18	39.1	0.4	0.9	1.5
	> 60	13	30.2	2	4.3	0.001	10.6	0.2
	< 40	15	16.9	37	61.7	< 0.0001	31.7	0.1
TT	40–60	48	53.9	15	25.0	0.0007	12.3	3.5
	> 60	26	29.2	8	13.3	0.03	5.1	2.7

IL-6 GG was more frequent among controls than NODAT in < 40 years old individuals, *p* ≤ 0.0001; while, the frequency was more in NODAT than controls among 40–60 years old individuals, *p* = 0.03. IL-6 CG was more frequent among control than NODAT, *p* = 0.0001 in < 40 years individuals; while, it was significantly associated with the disease in > 60 years old, *p* = 0.002.

In the same manner, IL-6 CC showed to more frequent in control in < 40 years old individuals, *p* = 0.0001; while the same genotype showed to be significantly associated to the disease in > 60 years old individuals, *p* = 0.005.

IL-4 TT was more frequent in control than NODAT among < 40 years old individuals, *p* = 0.01; while it 699 was more in NODAT than control among > 60 years individuals, *p* = 0.03. In the same manner, IL-4 TC was significantly increased in control subjects in < 40 years old individuals, *p* ≤ 0.0001; while, its association was deviated towards NODAT in 40–60 and > 60 years old individuals, *p* = 0.04, < 0.0001 respectively.

The same was true in IL-4 CC which was significantly increased in controls, *p* ≤ 0.0001; while its association was shifted towards NODAT in 40–60 years individuals, *p* = 0.006.

In the same manner, IFNG AA also showed the same association with the disease in which it has been highly associated in controls in < 40 and its association was deviated towards NODAT in 40–60 and > 60 years old, *p* = 0.0002, 0.04 and 0.02 respectively.

Interestingly, the same association was repeated among IFNG AT and TT. Where genotypes were highly associated with control subjects in < 40 and deviated then towards NODAT in older ages, *p* ≤ 0.0001, 0.01, 0.002, < 0.0001 and 0.03 respectively.

Interestingly, the same manner has been followed up in TGFB CC, TC and TT genotypes. All were highly associated with controls in < 40 years old, *p* ≤ 0.0001, 0.0001 and < 0.0001 respectively.

The genotypes association was deviated towards NODAT occurrence in 40–60 and > 60 years individuals, *p* = 0.03, 0.0002, 0.001, 0.0007 and 0.03 respectively. The above scenarios indicate lack of independent association of IFNG, IL-4, IL-6association of our key cytokines were not independent as they were following the old age association manner.

Likewise, IL-4 TC was more frequent among older participants than 40 years in control, *p* < 0.0001, while its distribution was shifted in older age groups towards NODAT, *p* = 0.04, (among 40–60 years of age) and *p *< 0.0001 among > 60 years of age, Table 5. FNG AA, AT, TT was more dominant in controls less than 40 years, *p* = 0.0002, *p *< 0.0001, *p* < 0.0001 respectively. Its genotype frequencies were shifted towards NODAT in 40–60 years of age groups, *p* = 0.04, 0.01, 0.04 respectively (Table 5). In the same order, the genotype distributions of IFNG were predominantly more in NODAT than the control with > 60 years of age, *p* = 0.02, *p* = 0.02, *p* = 0.009 respectively (Table 5).

The distribution of TGFB followed almost the same pattern as IFNG. The distribution of all genotypes were predominantly more in control among those older than 40 years, *p* < 0.0001, *p* = 0.001, *p* < 0.0001. The distributions were shifted towards dominance in NODAT among older age groups, *p* = 0.03, *p* = 0.4, *p* = 0.0007 in 40–60 years of age and *p* = 0.002, *p* = 0.001and *p* = 0.03 among > 60 years of age groups (Table 5). The frequency of the above cytokines was investigated according to their HLA class I -A and -B and class II DR but no statistical association was noted.

## Discussion

The presence of NODAT has increasingly emerged as an important factor in the long-term outcome of KTRs, including mortality^59^. In the current study, the patient demographics of NODAT and control cohorts were indistinguishable. There were more males than females in both cohorts. This was similar to several other studies which reported that organ-recipients were mainly males. It is possible this reflects a gender bias in the incidence of transplant-related pathologies^60,61^. However, some differences were noted, patients with chronic HCV and CMV infections were significantly more prevalent amongst the NODAT group. Furthermore, in our study, age appears to be an important contributing factor in the development of NODAT. More KTRs < 40 years of age were found in the control group, while those > 40 years of age were more susceptible to NODAT. These findings are in line with several reports from Europe and the USA^1–3,62,63^. There was a 90% increase of relative risk in kidney transplant patients aged 45–59 years and a 160% increase in patients older than 60 years compared to patients between 18–44 years old^63^. However, our data did not support findings concluded from studies from Egypt, Bahrain, Saudi Arabia, and Iran^9–11,64^. This could be explained by transracial differences or a lack of large systematic studies.

Associations of IL6 GG, IL-4 CC, IFNG TT and TGFB TT genotypes were superseded by age group and bio-physiology of patients. This might confirm identified differences in innate immune system in older KTRs compared to younger ones^65^.

Interestingly, patients with IL-6(GG), IFN-G (TT), and TGFB (TT) genotypes, which were associated with high protein production, were significantly higher in the NODAT cohort. While, IL-4(CC), related to low protein production, was significantly associated with the disease. On the contrary, HCV infection has been shown to be associated with IFNG and the development of type 2 diabetes mellitus in general population^66^.

Previous studies have suggested that asymptomatic CMV infection and CMV disease are independently associated with the development of NODAT^67^, while other studies reported that CMV was not a risk factor for NODAT^62,63,68^. In pretransplant evaluation we found a significantly higher number of patients with positive CMV IgG in NODAT cohort, while CMV IgM was comparable between both cohorts. Interestingly, there were significantly more patients with post-transplantation CMV viremia in control cohort, especially among IL-6 (GG), IL-4 (TC) and TGFB (TC), but no statistical association was found between IFNG and CMV in either cohorts. This could be explained by the use of routine antiviral chemoprophylaxis among our patients after transplant which nullified CMV as a risk for NODAT in our cohort. The significant association of genetic polymorphisms in IL-6, IFNG, TGFB in NODAT could be due to Th1 mediated immunity which occurs as a result of deviation of SOCS/T-reg Fox P3 balance. Our results support a previously reported deviation in SOCS/T-reg Fox P3 balance causing immune dysregulation by IFN-γ. The association of IL-6 (GG), IFNG (TT) and TGFB (TT) high protein producers and IL4 (CC), low IL-4 protein producer might go some way to explain these findings.

KTRs gender did not appear to effect genetic susceptibility of either IL-6 G (− 147) C, IL-4 C (− 590) T, IFNG, and TGFB genotypes. However, there was a clear association with age and the distribution of IL-6 G (− 147) C, IL-4 C (− 590) T, IFNG, and TGFB genotypes among NODAT and control groups. The associations of IL6 G (− 147) G, IL-4 C (− 590) C, IFNG TT and TGFB TT were superseded by age group and bio-physiology of patients. In short, IL6 G (− 147) G, IL-4C (− 590) C, IFNG TT and TGFB TT were not independently associated to NODAT. The current data might indicate that the latter immune signature complex can provide a clue to the pathogenesis of NODAT. Although throughout our induction procedure, we aimed to suppress T-cells, thereby reducing the chance of kidney rejection.

## Conclusion

The ability to perform noninvasive testing to transplant recipients will provide tools to identify patient risk of NODAT and the individualization of immune suppression regimens to improve outcomes after transplantation. The pathogenesis of NODAT is Th-1 cell-mediated variations, while IFNG, IL-4, TGF-β1 and IL-6may play a crucial role in that mechanism. Interestingly no association was found between the above cytokine genes and well-established HLA loci for NODAT. The latter rules out the autoimmune nature of NODAT.

Tailoring of immunosuppressive agents may be used to target patients according to their genetic makeup. The results of our research might provide a suitable platform for a larger multicenter study, focusing on the Arab population, to evaluate the role of cytokine genes in NODAT to confirm our findings and better understand the prevalence of NODAT in KTR patients.
